# Supplementary material for: COZOID: contact zone identifier for visual analysis of protein-protein interactions
Source: BMC Bioinformatics. 2018 Apr 6;19:125. doi: 10.1186/s12859-018-2113-6 (PMC5889581; doi:10.1186/s12859-018-2113-6)
Supplement: Supplementary file 2 — Software build. Executable binary file of the software tool. (ZIP 118,784 kb) [file 12859_2018_2113_MOESM2_ESM.zip › cozoid/java/docs/beansbinding-1.2.1-doc.zip!/org/jdesktop/el/impl/parser/AstNull.html]

AstNull (Beans Binding)


---


|  |  |  |
| --- | --- | --- |
| **Overview** \| **Package** \| **Class** \| **Use** \| **Tree** \| **Deprecated** \| **Index** \| **Help** | |  |
| **PREV CLASS**   **NEXT CLASS** | **FRAMES**    **NO FRAMES**     **All Classes** |  |
| SUMMARY: NESTED \| FIELD \| CONSTR \| METHOD | DETAIL: FIELD \| CONSTR \| METHOD |  |


---


## org.jdesktop.el.impl.parser Class AstNull

```
java.lang.Object
  org.jdesktop.el.impl.lang.ELSupport
      org.jdesktop.el.impl.parser.SimpleNode
          org.jdesktop.el.impl.parser.AstNull
```

**All Implemented Interfaces:**: Node

---

``` public final class AstNull extends SimpleNode ```

---

**Field Summary**

**Fields inherited from class org.jdesktop.el.impl.parser.SimpleNode**

`children, id, image, parent`


**Constructor Summary**

`AstNull(int id)`


| **Method Summary** | |
| --- | --- |
| `java.lang.Class` | `getType(EvaluationContext ctx)` |
| `java.lang.Object` | `getValue(EvaluationContext ctx)` |

**Methods inherited from class org.jdesktop.el.impl.parser.SimpleNode**

`accept, dump, getImage, getMethodInfo, invoke, isReadOnly, jjtAddChild, jjtClose, jjtGetChild, jjtGetNumChildren, jjtGetParent, jjtOpen, jjtSetParent, setImage, setValue, toString, toString`

**Methods inherited from class org.jdesktop.el.impl.lang.ELSupport**

`coerceToBoolean, coerceToCharacter, coerceToEnum, coerceToNumber, coerceToNumber, coerceToNumber, coerceToNumber, coerceToString, coerceToType, compare, containsNulls, equals, isBigDecimalOp, isBigIntegerOp, isDoubleOp, isDoubleStringOp, isLongOp, isStringFloat, throwUnhandled, toFloat, toNumber`

**Methods inherited from class java.lang.Object**

`clone, equals, finalize, getClass, hashCode, notify, notifyAll, wait, wait, wait`

**Constructor Detail**

### AstNull

```
public AstNull(int id)
```


**Method Detail**

### getType

```
public java.lang.Class getType(EvaluationContext ctx)
                        throws ELException
```

:   **Specified by:**: `getType` in interface `Node` **Overrides:**: `getType` in class `SimpleNode`
:   **Throws:**: `ELException`

---


### getValue

```
public java.lang.Object getValue(EvaluationContext ctx)
                          throws ELException
```

:   **Specified by:**: `getValue` in interface `Node` **Overrides:**: `getValue` in class `SimpleNode`
:   **Throws:**: `ELException`


---


|  |  |  |
| --- | --- | --- |
| **Overview** \| **Package** \| **Class** \| **Use** \| **Tree** \| **Deprecated** \| **Index** \| **Help** | |  |
| **PREV CLASS**   **NEXT CLASS** | **FRAMES**    **NO FRAMES**     **All Classes** |  |
| SUMMARY: NESTED \| FIELD \| CONSTR \| METHOD | DETAIL: FIELD \| CONSTR \| METHOD |  |


---
